# Supplementary material for: Are carbon emissions trading and green financial instruments synergistic? -Comprehensive quantitative research based on content analysis
Source: PLoS One. 2024 Mar 7;19(3):e0298601. doi: 10.1371/journal.pone.0298601 (PMC10919601; doi:10.1371/journal.pone.0298601)
Supplement: S1 Data — (ZIP) [file pone.0298601.s001.zip › Data/地级市Z_F工作报告.zip!/╕╩╦α/└╝╓▌╩╨/└┤╫╘╣1⁄2╓┌║┼í╛┬φ┐╦╩2╛▌═°í┐_2016.docx]

各位代表：

现在，我代表市人民政府向大会作报告，请与《兰州市国民经济和社会发展第十三个五年规划纲要（草案）》一并审议，并请各位政协委员和其他列席人员提出意见。

一、“十二五”时期经济社会发展回顾

过去五年，全市上下在市委的正确领导下，认真落实省委、省政府做大做强做美兰州的系列部署和要求，抓住机遇乘势而上，凝聚力量攻坚克难，在抓发展、治污染，拓空间、畅交通，强管理、提效能，保民生、促和谐等重点工作上进行突破，全面完成“十二五”规划各项约束性指标，基本实现预期性目标，开创了兰州经济社会发展的新局面。特别是办成了一批打基础、利长远、惠民生、增信心的大事：

——大气污染治理取得历史性突破，打造了国内外瞩目的“兰州蓝”，成为全国重点监测城市中环境空气质量指数下降最快的城市，稳定退出全国十大重污染城市行列，得到国家的充分肯定并总结推广了“兰州经验”，在巴黎世界气候大会上荣获“今日变革进步奖”。我市被中央电视台“中国经济生活大调查”评为2015年度“中国十大幸福城市”第五名。

——获批建设国家级兰州新区，秦王川这片昔日的旱塬之地，正在成为西北地区重要经济增长极、国家重要产业基地、向西开放重要战略平台、承接产业转移示范区。

——获批开建城市轨道交通，1号线一期工程加快建设，2号线一期项目启动实施，全市人民的地铁梦即将变成现实。

——开工建设新的水源地工程，将从黄河上游刘家峡水库自流引水，根本上解决广大群众喝上放心水的问题。

——建成运营兰州新区综合保税区，实现海关特殊监管区零的突破，成为兰州扩大开放、发展外向型经济的新平台。

——实现全市整体脱贫，减少贫困人口23.84万人，贫困发生率由25%下降到4%以下。

——成功举办兰州国际马拉松赛，成为国内马拉松十大金牌赛事之一和“100强赛事”第四名，展示了城市形象，弘扬了城市精神，丰富了城市内涵，激发了全市人民的归属感和自豪感。

五年来，我们聚焦中心带动，奋力加速发展，实现了综合经济实力的新跨越。全市生产总值达到2095.99亿元，比2010年接近翻一番，在全省的首位度由26.7%提升到30.86%。人均生产总值从4704美元提高到8738美元，增长85.76%。固定资产投资年均增长26.76%，达到1803.75亿元，五年完成6841.78亿元，是“十一五”的3.03倍。社会消费品零售总额年均增长14.14%，达到1152.15亿元，是2010年的2.11倍。地区财政总收入和一般公共预算收入达到593.42亿元和185.19亿元，分别是2010年的1.95倍和2.55倍。非公经济增加值年均增长22.3%，达到1187亿元，占经济总量的比重从40%提高到56.6%。全市金融机构各项存款余额、贷款余额和住户存款余额达到7945.82亿元、7227.96亿元和2627.1亿元，分别是2010年的2.46倍、3.06倍和2.03倍，实现直接融资255.59亿元，是2010年的48倍。获批全国节能减排财政政策综合示范城市、红古区资源枯竭城市转型、七里河区老工业基地搬迁改造试点、全国质量强市示范城市等40多个国家和省级政策平台，五年向上争取各类资金181.51亿元。

五年来，我们培育发展多元支柱产业，大力调整优化产业结构，实现了产业转型升级的新跨越。坚持一手改造升级传统产业，一手培育发展战略性新兴产业，三次产业结构由3.07∶48.09∶48.84调整为2.68∶37.34∶59.98，第三产业比重提高11.14个百分点。制定实施《关于发展培育多元支柱产业的意见》和《产业布局与园区发展规划》，实施工业项目189个，新增工业产值326.7亿元，全市工业增加值达到535.04亿元，年均增长11.03%。启动107户工业企业“出城入园”，兰石集团、青岛啤酒等48户企业建成投产。大力发展循环经济，成为国家“城市矿产”示范基地。推进兰白科技创新改革试验区建设，制定出台“1+8”科技创新改革政策，建成兰州新区产业孵化大厦等科技企业孵化器27个，创办兰州科技成果交易会，运营兰州科技大市场，组建7个产业投资基金，战略性新兴产业增加值达到175亿元，占生产总值的比重为8.35%。制定“1+10”鼓励“大众创业、万众创新”政策措施，培育船说创业咖啡、得力帮生物化工、“1898”等新型众创空间66家。调整优化城市商贸物流布局，规划建设瑞鑫、毅德、北龙口等大型物流园区。成功举办国际跨境电商物流大会，全市电商企业超过2000家，交易规模达到722亿元，实现跨境电商零的突破，成为国家电子商务示范城市。编制实施《兰州都市文化产业区规划纲要》，加快打造“中国黄河文化体验之都、丝绸之路文化产业名城”，推进创意文化产业园、青城古镇、河口古镇等重点项目建设，承办中国金鸡百花电影节，文化产业增加值年均增长26.08%，达到60.91亿元，是2010年的3.1倍。接待游客人数达到4121.26万人次，旅游总收入达到334.56亿元，分别是2010年的4.6倍和5.3倍。中川机场旅客吞吐量突破800万人次，国际地区旅客吞吐量突破20万人次，分别是2010年的2.22倍和20倍。

五年来，我们完善城市功能，提升城市形象，实现了打造宜居宜业宜游魅力城市的新跨越。编制并获批实施第四版城市总体规划，城市建设跳出河谷、拉开框架，东部科技城等片区融入城市建设范围。中心城区控制性详细规划和乡镇总体规划实现全覆盖。城市规划展览馆建成开馆。西客站、兰新高铁和中川城际铁路建成运营，铁路交通跨入高铁时代。轨道交通1号线一期工程完成投资109.37亿元，打通国内首条下穿黄河地铁隧道。构建城市“139”骨干路网体系，南山路建成通车，北环路加快建设，拓建和改造城市主次干道92条。建成“上跨下穿”工程4个。新建人行过街天桥45座。完成小街巷改造1022条。新建黄河大桥3座、在建2座。开通公共自行车租赁系统和黄河水上公交。开工建设保障性住房13.91万套。全面整治黄河两岸违法建设、环境卫生和各种经营场所，建成健身步道20公里，黄河风情线环境面貌明显提升。改造提升五泉山、白塔山、兰山、雁滩和金城5大公园，新建马拉松主题公园、雁滩湿地公园、黄河湿地公园，新增城市绿地514公顷。完成污水全收集全处理工程，污水处理率由60%提高到95%。统筹实施餐厨垃圾、生活垃圾、建筑垃圾处理等项目建设。改造“三不管”楼院289个。城市道路机械化清扫率由30%提高到90%。推进“数字城市”向“智慧城市”转型。强化城市应急管理体系建设，建成15个应急避难场所。实施“凡煤必改、应改尽改”的“换血式”煤改气工程，改造主城区燃煤锅炉1901台、8240蒸吨，实现燃煤锅炉及其污染“双清零”。在全国率先开展政府环境审计试点工作，环境能源交易工作走在全国前列。2015年城区空气质量优良天数达到252天，比2013年增加59天，PM10、PM2.5年均浓度比2013年分别下降21.6%和22.4%。

五年来，我们拓展城市空间，促进产城融合，实现了兰州新区开发建设的新跨越。兰州新区累计完成投资1447.67亿元，核心区120平方公里的水、电、路、气等基础设施基本形成，建成道路116条450公里，兰秦快速通道建成通车。围绕六大主导产业，累计引进产业项目281个，兰石重装、科天化工、四联光电、亚太伊士顿电梯、陇星热能等44个项目建成投产，绿地智慧金融城、正威电子信息产业园、长城梦世界旅游文创园、西部国际文商旅综合生态产业区等164个产业项目加快建设。“飞地经济”园区启动建设。加快产城融合发展，市体育学院、兰州新区舟曲中学、兰州十一中新区分校、兰炼一小新区分校、兰州实验幼儿园新区分园建成投用，兰州六十一中新区分校、省人民医院新区分院等加快建设。完成造林绿化18.5万亩，建成2号生态湖、百花公园、迎宾公园，秦王川国家湿地公园加快建设，初步形成生态新区景观体系。兰州新区生产总值达到125.53亿元，是获批建区时的2.23倍。高新区、经济区增容扩区取得重大突破，生产总值分别达到178.77亿元和225.98亿元，是五年前的2倍和2.41倍。

五年来，我们坚持“双联”和扶贫攻坚深度融合，全力推进农村发展，实现了城乡统筹发展的新跨越。扎实开展精准扶贫、精准脱贫，制定实施“1+21”精准脱贫方案，五年整合投入财政资金182亿元，农村居民人均可支配收入年均增长14.58%，达到9621元，比2010年翻一番，成为农业综合生产能力极大提高、农村面貌极大改善、农民生活极大提升的时期。农业特色富民产业明显提质增效，高原夏菜、中药材、玫瑰、百合和规模养殖等特色产业发展壮大，“兰州百合”荣获2015意大利米兰世博会优质特产奖和特色产业推广贡献奖。市级以上农业龙头企业达到133家，农民专业合作社达到3518家。农村环境发生历史性变化，新建农村公路2346公里，建制村通畅率达到100%。连续实施农村饮水安全工程，解决50.59万人饮水安全问题，完成39座大中型泵站改造。新建标准化卫生室47个、文化活动室205个、全民健身场地607个，实现全市行政村全覆盖。建成省级美丽乡村示范村21个、市级美丽乡村示范村40个。改造农村危房22440户。全市城镇化率由76.28%提高到80.95%。

五年来，我们突出民生导向，顺应广大群众新期待，实现了社会建设和民生改善的新跨越。着力抓好就业、就学、就医、收入、住房和社会保障等民生实事，累计用于民生方面的投入达到354.3亿元。全面建成小康社会综合实现程度达到86.77%，提高12.35个百分点。城镇新增就业50.1万人，实现劳务输转164.5万人。城镇居民人均可支配收入年均增长12.6%，达到27088元，比2010年接近翻一番。基本建立覆盖城乡的社会保障体系，城乡居民基础养老金分别提高90.9%，城乡低保补助分别提高85%和188%，城乡医保补助各提高90%。城乡办学条件大幅改善，引进建成北京实验二小兰州分校，新建城乡公办幼儿园124所、寄宿制学校167所、标准化学校209所，全面消除中小学D级危房。医疗卫生服务体系不断健全，新增医院床位3000张，县级以下医院、卫生院、卫生所全部实现标准化。三维数字社会服务管理系统被确定为国家行业标准在全国推广。“虚拟养老院”服务模式被称为我国养老事业“破题之举”，服务人数达到24万人。建成残疾人综合服务中心和托养就业康复中心。公益性文化设施全部免费开放。数字影院覆盖远郊三县一区。群众体育工作走在全国前列。建立“平价肉菜进社区”和冬春蔬菜储备制度。实施7939家餐饮单位“明厨亮灶”工程，建成九州“主食厨房”一期。树立了“兰州好人”等文明创建品牌，“凡人善举、和你一起”活动入选中宣部创新案例，永登县苦水镇荣获“全国文明村镇”称号。开展了海军“兰州舰”命名入列十周年系列纪念活动，荣获全国双拥模范城“八连冠”。民族宗教工作“三个共同、两个提升”活动深入开展。安全生产四项控制指标持续下降。在全国率先建设环城反恐治安卡口检查站。八类严重暴力案件占刑事全案的比重从12.6%下降到3.6%，2014年、2015年命案侦破率达到100%。

五年来，我们统筹向东承接和向西开放，融入丝绸之路经济带建设，实现了对外开放的新跨越。制定参与“一带一路”建设实施方案，全力打造丝绸之路经济带核心节点城市。举办了丝绸之路经济带（兰州新区）发展高层论坛、丝绸之路经济带智库论坛、国际清真科技产业发展论坛等系列节会，营造了积极参与丝绸之路经济带建设的浓厚氛围。在全国省会城市中率先加入上海合作组织睦邻友好委员会。开辟首尔、曼谷、迪拜、第比利斯、圣彼得堡等国际航点16个。缔结友好合作交流城市8个。开通“兰州号”中亚国际货运班列和中欧国际货运往返班列，并实现常态化运营，累计开行26列、1140车。启动实施兰州国际港务区建设，东川铁路货运中心投入运营。全市进出口总额由18.79亿美元增长到51亿美元，面向丝绸之路经济带沿线国家的占到四分之一。五年招商引资到位资金6022.11亿元，是“十一五”的8倍，引进“三个500强”企业42家。深入开展人文交流，组织群众文艺团队赴俄罗斯、英国参加国际民间交流艺术节，10多个国家和地区来兰开展文化艺术交流展演活动，1100多名中西亚学生在兰留学。

五年来，我们全面深化改革，完善体制机制，实现了发展环境优化的新跨越。持续深化行政审批制度改革，取消、调整和下放行政审批事项255项，市级政府部门行政审批事项减少到201项。市政务服务中心全面推行重大项目并联审批和代办服务模式，提高了为民服务水平。建成政务电子监察系统，实现对行政审批、行政处罚、公共资源交易等事项全方位监控。全面推开“一照一码”商事制度改革，极大释放市场活力，全市市场主体累计达到27.92万户，比2010年翻一番。深化财政预算改革，全面推行预算内外收支统管和零基预算。金融体制不断完善，投融资市场发育程度明显提高，组建运营兰州农商银行，新引进金融机构8家，扶持6家企业上市融资，11家中小企业在新三板挂牌上市。组建兰州投资、交通、能源、工业、农业、文化等资本运营平台。建立市公共资源交易中心。基本完成农村土地确权登记。在全国省会城市率先开展不动产统一登记和发证工作。

五年来，我们着力加强政府自身建设，大力转变工作作风，实现了行政效能提升的新跨越。群众路线教育实践活动和“三严三实”专题教育深入开展，解决了一批“四风”和不严不实的突出问题。依法接受市人大及其常委会监督，及时报告工作，认真执行决议决定，积极配合开展执法检查、代表视察、专项调研和专题询问，办理人大代表建议1070件。自觉接受市政协民主监督，积极采纳政协委员调研成果和协商建议，办理政协委员提案1960件。提请市人大审议地方性法规22部，制定政府规章45部、修订规范性文件83件。着力推进依法行政和政务公开，编制运行“五张清单一张网”。重视群众来信来访和网民诉求办理答复工作，人民网留言回复率排全国省会城市前列。从严落实中央“八项规定”和国务院“约法三章”，市级“三公经费”支出下降73.78%。配合开展了经济责任履行和机构编制等重大审计工作。落实党风廉政建设主体责任，做到了“两手抓两手硬、双促进双落实”。

刚刚过去的2015年，面对宏观经济下行压力的影响，我们突出抓好稳增长、促改革、调结构、惠民生、防风险等重点工作，全市经济社会发展稳中有进、稳中有新、稳中有好。生产总值增长9.1%，分别高于全省、全国1个和2.2个百分点；城镇居民人均可支配收入、农村居民人均可支配收入分别增长10.5%和12.3%，明显高于经济增速；一般公共预算收入增长21.57%，排全国省会城市第1位；一般公共预算支出增长22.85%；物价上涨1.3%，低于控制目标；城镇登记失业率为1.76%；全面完成为民兴办的24件实事。

各位代表，“十二五”是我市经济发展加速、产业结构优化、发展空间拓展、创新活力增强、开放开发加快的五年，也是城乡面貌变化最大、广大群众得到实惠最多的时期之一。这五年所取得的成就，是省委、省政府和市委坚强领导的结果，是全市人民共同奋斗的结果。在这里，我代表市人民政府，向各位代表、政协委员和全市各族人民，向各民主党派、工商联、人民团体和社会各界人士，向离退休老同志，向驻兰解放军指战员、武警官兵和公安民警，向新闻媒体和广大网友，向所有关心、支持和参与兰州建设发展的同志们和朋友们表示崇高的敬意和衷心的感谢！

回顾过去五年的工作，我们也清醒地认识到，我市经济社会发展中还存在一些突出矛盾和问题。传统产业改造升级任务繁重，新兴产业尚处在培育壮大之中，需要加快转方式、培育新动能；城市功能和管理还存在不少短板，基础设施建设和公共服务供给不足，城市整体环境与快速成为国家向西开放重要战略平台的地位不相适应；虽然在大气污染治理上取得显著成效，但生态环境依然脆弱，综合承载能力不强，走绿色发展之路任重道远；广大群众关心的教育、医疗、养老、收入、户籍、食品药品安全等方面问题较多，各类公共安全、生产安全事故时有发生，尤其是2014年、2015年连续发生自来水异味问题，教训极其深刻；政府职能转变和机关作风还不完全适应新常态的要求，行政效能和执行力有待进一步提高，个别政府工作人员发生严重违纪违法问题，影响了政府形象。为此，我们将在工作中更加突出问题导向，努力破解难题、补上短板。

二、“十三五”发展的主要目标任务

根据《中共兰州市委关于制定国民经济和社会发展第十三个五年规划的建议》，市政府在广泛征求意见、集中各方智慧、充分研究论证的基础上，编制了《兰州市国民经济和社会发展第十三个五年规划纲要（草案）》，提出了“十三五”全市经济社会发展的指导思想、发展目标、主要任务和重大支撑。今后五年发展的重点是，充分发挥省会中心城市的综合优势，着力推动大都市、大产业、大枢纽、大物流、大市场、大平台建设，进一步做大做强做美兰州。

一、关于经济增长预期目标。《规划纲要》提出，“十三五”时期全市经济年均增长8%，高于全国全省平均水平，到2017年生产总值比2010年翻一番，2020年超过3000亿元，人均生产总值达到76000元左右。确定这一目标，充分兼顾了目前发展实际和长远发展需要，也力求体现在全省的中心带动作用，逐步缩小与全国的发展差距。省上确定“十三五”时期经济增长为年均7.5%，我市8%的年均增速高于全省0.5个百分点，按照2015年生产总值2095.99亿元测算，实现2017年比2010年翻一番和2020年超过3000亿元的目标是完全可以的。

二、关于创新驱动发展。《规划纲要》把创新摆在更加突出的位置，提出到2020年研发经费占生产总值比重达到2.54%，战略性新兴产业增加值占生产总值的比重达到16%以上，科技对经济增长的贡献率达到60%，这既是在资源环境约束趋紧情况下保持经济中高速增长的现实需要，也是推进经济转型升级、持续提升发展质量效益的必然选择。兰州作为省会中心城市，各种创新资源和要素比较集中，特别是随着兰白科技创新改革试验区建设步伐加快，兰州新区、高新区和经济区科技创新平台作用逐步放大，科技支撑经济社会发展的能力会持续增强。只要我们坚定不移大力支持大众创业、万众创新，积极营造更加精准有效的创新政策制度环境，着力强化和发挥企业创新主体地位和主导作用，实现上述预期目标是有把握的。

三、关于统筹协调发展。省委关于“十三五”发展的《建议》提出，“推动中部经济区率先突破发展，强化兰州在全省的辐射、带动和支撑作用”。根据这一要求，市委《建议》强调在“十三五”时期着力推进大都市建设，打造全国有影响力的现代化中心城市。为此，《规划纲要》突出区域协调发展问题，明确了加快“大兰州”经济区建设，促进“兰西城市群”互动开放，加强联合走西口的跨省域发展等重点工作，尤其要构建以兰州为中心的半小时和一小时经济圈，促进与周边城市、重点城镇的同城化发展，在全省的辐射、带动和支撑作用进一步增强。在城乡协调发展上，重点是以新型城镇化建设为方向，优化空间布局结构，着力构建“主城区—兰州新区—县（区）—特色小城镇—美丽乡村”一体化发展的城乡体系，把更多的公共资源投向农村，形成以城带乡、城乡一体的发展新格局，常住人口城镇化率达到85%以上。

四、关于生态保护与环境建设。《规划纲要》提出，“十三五”时期单位地区生产总值能耗、用水量、二氧化碳排放量分别下降15%、50%、18%，地表水质量好于三类水体比例达到100%，全市森林覆盖率达到18%，城区绿地率达到35%以上，绿化覆盖率达到40%以上，建成国家园林城市。基于以上目标，我们将全面推进低碳城市建设，围绕2025年实现碳排放达峰的目标，积极促进能源结构调整，使非化石能源占一次性能源消费比重保持在18%以上，煤炭消费比重降至60%以下；持续改善空气环境质量，加快建立大气污染防治标准体系，推动大气污染治理制度化、法治化、常态化，到2020年城区空气质量达标率超过80%，PM2.5浓度下降到40微克/立方米以下,力争好于全国平均水平；着力发展循环经济，建立农业、工业、服务业和社会四位一体的循环经济发展体系；推进国家生态文明先行示范区建设，加强生态绿化，建立生态补偿机制，实现绿色发展、循环发展、低碳发展。

五、关于城乡居民收入。《规划纲要》提出，“十三五”时期城乡居民人均可支配收入年均分别增长10%和10.2%，城乡收入比缩小到2.8∶1以下。一是考虑了居民收入增长必须与经济增长同步或高于经济增速的政策要求。二是考虑了全面建成小康社会统计监测指标体系的达标需求，只有保持较高的增长速度，才能保证人民生活水平不断提升，尽早达到全面小康社会统计监测指标标准。三是考虑了新型城镇化加速推进带来的增长带动，目前我市已转入全面建设小康社会阶段，随着扶贫攻坚力度不断加大、产业项目持续推进实施、基础设施加快完善、社会保障体系不断健全，将有效带动城乡居民收入增长。

六、关于文化旅游产业发展。《规划纲要》提出，今后五年建设9个在全国有影响力的文化旅游景点景区，到2020年文化产业和旅游产业增加值分别占到生产总值的8%和5%以上，初步成为全市经济的支柱性产业。这是适应新常态、构建“大三产”格局的需要，也是挖掘和培育新经济增长点的重要举措。兰州作为古丝绸之路上的商埠重镇和“黄河之都”，具有独特丰富的文化旅游资源，“十二五”以来，借助省上建设华夏文明传承创新区的有利契机，我市确定并正在实施一批大项目、好项目，只要我们抓好项目这个支撑，推动文化与旅游、体育、农业等融合发展，延伸文化旅游产业链，提升文化旅游价值链，就能够把文化旅游产业培育成经济转型升级的支柱产业。

七、关于对外开放发展。《规划纲要》提出，今后五年全市进出口总额年均增长8%，其中与丝绸之路经济带沿线国家贸易额年均增长10%。随着国家“一带一路”战略的实施，兰州作为丝绸之路经济带核心节点城市功能日益凸显，正在从传统的内陆城市成为向西开放的前沿，我们抓住这个最大机遇，在互联互通、人文交流、对外贸易、招商引资等方面取得了积极成效。“十三五”时期，将通过建设国际港务区、打造综合交通枢纽、深化国际交流合作、建设丝路信息走廊、推动区域合作发展等一系列措施，构建与中西亚及欧洲商贸物流、产业合作、信息服务、人文交流的大平台，打造国家向西开放的新高地，使兰州“引进来”、“走出去”的双向开放水平得到新的提升。

八、关于基本公共服务。《规划纲要》提出，在教育方面，将持续加大投入，促进均衡优质发展；在就业方面，将坚持创业带动就业，大力扶持和发展众创空间，进一步培植就业增量；在社会保障方面，将突出养老保障体系的构建和完善，建设5个以上上千张床位的医养结合养老基地，养老设施覆盖95%以上乡镇和70%以上行政村；在公共文化体育服务方面，将实现市、县、乡、村四级公共文化设施标准化和体育场地全覆盖；在健康城市建设方面，市级综合医院全部达到三级甲等医院标准，市级专科医院全部达到
